# Machine learning–XGBoost analysis of language networks to classify patients with epilepsy

**DOI:** 10.1007/s40708-017-0065-7

**Published:** 2017-04-22

**Authors:** L. Torlay, M. Perrone-Bertolotti, E. Thomas, M. Baciu

**Affiliations:** 1 CNRS LPNC UMR 5105, Univ. Grenoble Alpes, 380000 Grenoble, France; 2 Laboratoire INSERM U1093, Université de Bourgogne, 21000 Dijon, France; 3 LPNC, UMR CNRS 5105, BSHM, Université Pierre Mendès-France, BP 47, 38040 Grenoble Cedex 09, France

**Keywords:** Language, Epilepsy, Atypical, Machine learning, ML, Extreme Gradient Boosting, XGBoost

## Abstract

Our goal was to apply a statistical approach to allow the identification of atypical language patterns and to differentiate patients with epilepsy from healthy subjects, based on their cerebral activity, as assessed by functional MRI (fMRI). Patients with focal epilepsy show reorganization or plasticity of brain networks involved in cognitive functions, inducing ‘atypical’ (compared to ‘typical’ in healthy people) brain profiles. Moreover, some of these patients suffer from drug-resistant epilepsy, and they undergo surgery to stop seizures. The neurosurgeon should only remove the zone generating seizures and must preserve cognitive functions to avoid deficits. To preserve functions, one should know how they are represented in the patient’s brain, which is in general different from that of healthy subjects. For this purpose, in the pre-surgical stage, robust and efficient methods are required to identify atypical from typical representations. Given the frequent location of regions generating seizures in the vicinity of language networks, one important function to be considered is language. The risk of language impairment after surgery is determined pre-surgically by mapping language networks. In clinical settings, cognitive mapping is classically performed with fMRI. The fMRI analyses allowing the identification of atypical patterns of language networks in patients are not sufficiently robust and require additional statistic approaches. In this study, we report the use of a statistical nonlinear machine learning classification, the Extreme Gradient Boosting (XGBoost) algorithm, to identify atypical patterns and classify 55 participants as healthy subjects or patients with epilepsy. XGBoost analyses were based on neurophysiological features in five language regions (three frontal and two temporal) in both hemispheres and activated with fMRI for a phonological (PHONO) and a semantic (SEM) language task. These features were combined into 135 cognitively plausible subsets and further submitted to selection and binary classification. Classification performance was scored with the Area Under the receiver operating characteristic curve (AUC). Our results showed that the subset SEM_LH BA_47-21 (left fronto-temporal activation induced by the SEM task) provided the best discrimination between the two groups (AUC of 91 ± 5%). The results are discussed in the framework of the current debates of language reorganization in focal epilepsy.

**Electronic supplementary material:**

The online version of this article (doi:10.1007/s40708-017-0065-7) contains supplementary material, which is available to authorized users.

## Introduction

Focal epilepsy is characterized by symptoms induced by lesion or dysfunction of a specific cerebral region, the ‘epileptic zone’ (EZ) [1]. Given the location of the EZ within or in the vicinity of language networks, patients are more or less impaired for language abilities but they clearly show reorganization of language networks based on functional plasticity [2, 3]. Compared to typical left hemisphere representation of language observed in the majority of healthy subjects [4], patients with epilepsy show a higher frequency of atypical language representation, with both inter- and intra-hemispheric reorganization (see [3] for a review). In addition, an important percentage of patients with epilepsy become resistant to anti-epileptic medication and surgery is undertaken to remove the EZ and stop seizures [5, 6]. In the pre-surgical phase, language mapping is required to identify language regions that must be preserved during surgery to avoid cognitive deficit. Mapping provides information in terms of language specialization, either at a hemispheric or at a regional level, as both levels provide specific patterns of reorganization. The global hemispheric specialization for language (and other cognitive functions) is clinically assessed with the Wada test [7, 8], while language networks are generally defined at an inter- and intra-hemispheric level with noninvasive methods such as functional MRI. Functional MRI maps language in patients with epilepsy by using a large variety of tasks and protocols [9–17] even if there is no clear consensus on the most appropriate task or panel of tasks to be used. In clinical practice, phonological and lexico-semantic tasks are generally used to maximize the amount of relevant information for language network activity. The identification of language patterns, mostly atypical in patients, requires precision (in terms of language tasks and analysis of data) to correctly describe the reorganized networks and to avoid deficits after surgery.

In terms of data analysis, we generally determine (qualitatively/inspection or quantitatively/statistically) for a given task and a given patient, the language network and its functional hemispheric or regional lateralization [17]. This individual analysis has inherent limits linked to the high intra- and inter-patient variability of activation, giving rise to poor statistical power and subjective interpretation [18, 19]. These limits are particularly important in the case of patients who need surgery and show a high risk of postsurgical sequels if the region responsible for seizures is located in the vicinity of reorganized language networks, which are not correctly identified before surgery. In sum, there is a strong need to develop robust statistical and objective approaches to identify language networks in patients with epilepsy. Functional MRI is a very helpful tool to highlight activated regions but the information on this activation is not sufficient [20]. Specifically, this activation needs to be further processed and included into more robust statistical analyses, to obtain robust results allowing for a clearer distinction between patients with reorganized cognitive networks and healthy subjects with typical representations of cognitive functions. Due to the lack of statistical power when trying to validate differences between single-patient fMRI measures and a group of heathy subjects, robust statistical methods should be involved.

The main objective of this study is to evaluate an objective method to distinguish patients and healthy people, based on language networks mapped with fMRI, and by using a machine learning (ML) approach. Previous results from a range of cognitive studies [21–24] showed successful use of ML classification. In patients with epilepsy, an ML approach based on a probabilistic regression method was used on fMRI data to evaluate the hemispheric specialization for language before surgery [18]. The authors showed successful classification (96%) with dissociation between typical (i.e., left hemisphere predominance) and atypical patterns of lateralization. Moreover, patients with atypical patterns (i.e., right hemisphere dominant or bilateral representation) were successfully identified (82%). An important advantage of the ML approach is that predetermined parameters (a priori threshold value settings as classically used in fMRI analysis) are not necessary, removing the subjective dimension of analyses and interpretations. Other authors have used ML on data concerning the integrity of white matter fibers to predict the surgical outcome in patients with epilepsy [25]. This approach was able to distinguish patients with epilepsy from normal controls with 80% accuracy, and predict the surgical outcome for patients, with 70% accuracy. ML classification requires input features or dimensions. In fMRI, these features are represented by the amount of the blood oxygen level-dependent (BOLD) signals in regions of interest or by lateralization indices, as used by [18]. The latter are calculated with BOLD signal values measured in homologues (right and left hemisphere) regions of interest. fMRI activity and BOLD signals strongly depend on psycholinguistic features such as language operation (phonology and semantic) and tasks used during fMRI assessment. Ideally, a fine-grained representation of language networks in patients implies the use of a panel of language tasks. This is difficult to apply in clinical practice due to practical reasons (short duration of the fMRI protocol, tasks should be easy to perform by patients). Consequently, a compromise has to be found between the amount of information in terms of language networks and pragmatic criteria inherent to work with patients. In this framework, it is accepted that the essential information concerning language networks in patients with epilepsy is obtained by using a phonological and semantic task (see, for instance, [3] and [15]).

In this current study, we applied a ML classifier, the *Extreme Gradient Boosting* algorithm (XGBoost) [26] in order to discriminate the fMRI from epileptic patients and healthy subjects. This particular method was chosen due to its significant advantages: (a) dealing with missing values, (b) requiring data scaling, (c) implying a computationally efficient variant of gradient boosting algorithm [27], (d) providing satisfactory results in ML competitions [28] and was successfully used in other studies and domains (see [29, 30]). Using XGBoost, we expected to identify robust patterns of language representation which are able to distinguish patients and healthy people. To our knowledge, there are no studies using XGBoost to objectively classify two populations based on their neurophysiological features. Specifically, we examined 55 participants who underwent fMRI and performed two language tasks—a semantic and phonological one, which activate classical language regions [15, 16, 31–33]. Based on the activation, we defined 20 features, as follows: five fronto-temporal (FT) regions (BA, Brodmann Area; BA21, BA 22, BA 44, BA 45 and BA 47), delineated in each (left, LH; right, RH) or both (bilateral; LH-RH) hemispheres, and for each language task (SEM, PHONO). We expect that differences between healthy subjects and patients with epilepsy would reveal atypical patterns of language representation in the damaged brains of patients. The atypical patterns might also reflect ‘sensitive-to-surgery’ regions that must be preserved during surgery to avoid language deficits.

## Material and methods

### Participants

We examined 55 participants: 16 patients with focal epilepsy and 39 healthy controls. Patients showed various anatomical locations of the EZ, and as indicated in Table 1, they were right- and left-handed. All were native French speakers and had normal or corrected-to-normal vision. Healthy volunteers had no history of neurological or psychiatric disorders. Participants gave informed written consent, and the study was approved by the local ethics committee (CPP no 09-CHUG-14, 04/06/2009).

**Table 1:** Demographic information of participants, patients (TLE, patient with epilepsy with left temporal lobe epilepsy) and healthy volunteers (controls)

	*N*	Age mean (SD)	Gender	Handedness
TLE	16	35.3 ± 11.1	9M–7F	1L–15R
Controls	39	26.5 ± 3.7	18M–21F	15L–24R

For each group, we mentioned the number of participants (*N*), the mean age and standard deviation (*SD*), the gender (*F*, female; *M*, male) and the handedness (right-handed, *R*; left-handed, *L*)

### Stimuli and tasks

The experimental protocol used during fMRI examination is described in detail in [15]. Two language tasks were used in two separate runs, a phonological (PHONO) and a semantic (SEM) task, each one also including a control visual condition (without language demands). Each task comprised ‘language’ and ‘control’ conditions. The PHONO language condition was performed with pseudo-words. Participants were instructed to detect a target phoneme (phoneme detection task). The SEM language condition of SEM run was performed using words with participants being instructed to judge whether items designated living or non-living entities (categorization task). The control condition was identical for the two runs and was performed using unreadable words (font Karalyn Patterson) with participants being instructed to judge the height of characters (visual detection task). Stimuli generated by the E-Prime software (E-prime Psychology Software Tools Inc., Pittsburgh, USA) were written in white ‘Courier New’ font size 40, centered on the middle of a black screen and lasted 2.5 s each.

### Functional MRI paradigm

A pseudo-randomized event-related fMRI paradigm was optimized [34] for 60 events, and 35 additional null events were used for each run (PHONO and SEM runs). The null events were added in order to provide an appropriate baseline measure [1] and consisted of a white fixation cross-displayed in the center of the black screen. The inter-stimulus interval was 2.5 s. The run duration was 8 min 40 s.

### MR acquisition

The experiment was performed in a whole-body 3T MR scanner (Bruker MedSpec S300) with 40 mT/m gradient strength at MR facility. For functional runs, the manufacturer-provided gradient-echo/T2*-weighted EPI method was used. Thirty-nine adjacent axial slices parallel to the bi-commissural plane were acquired in an interleaved mode. Slice thickness was 3.5 mm. During each run, the cerebral volume was measured 150 times. The in-plane voxel size was 3 × 3 mm (216 × 216 mm field of view acquired with a 72 × 72 pixel data matrix, reconstructed with zero filling to 128 × 128 pixels). The main sequence parameters were: TR = 2.5 s, TE = 40 ms, flip angle = 77°. To correct images for geometric distortions induced by local B0 inhomogeneity, a B0 field map was obtained from two gradient-echo datasets acquired with a standard 3D FLASH sequence (ΔTE = 9.1 ms). The field map was used during data processing. A T1-weighted high-resolution three-dimensional anatomical volume was also acquired, by using a 3D-modified driven equilibrium Fourier transform (MDEFT) sequence (field of view: 256 × 224 × 176 mm; resolution: 1.333 × 1.750 × 1.375 mm; acquisition matrix: 192 × 128 × 128 pixels; reconstruction matrix: 256 × 128 × 128 pixels).

### Spatial preprocessing of fMRI data

Data analysis was performed by using the general linear model, GLM [35] for event-related designs with SPM12 (Wellcome Department of Imaging Neuroscience, London, UK, www.fil.ion.ucl.ac.uk/spm) implemented in MATLAB (MathWorks Inc., Natick, MA, USA). Images were spatially preprocessed. First, the functional volumes were time-corrected with the 19th slice as reference (the acquired brain volume was composed of 39 slices) to correct artifacts caused by the delay of time acquisition between slices. Subsequently, all volumes were realigned to correct for head motion, by using a rigid body transformation. T1-weighted anatomical volume was co-registered to mean images created by the realignment procedure and was normalized within the MNI space. Anatomical normalization parameters were used for the normalization of functional volumes. Each functional volume was smoothed by a Gaussian kernel of 8 mm FWHM (Full Width at Half Maximum). Finally, time series for each voxel were high-pass filtered (1/128 Hz cutoff) to remove low-frequency noise and signal drift.

### Statistical analyses of fMRI data

Statistical analyses were subsequently performed on the preprocessed data. For each participant, each task (PHONO and SEM) was declared as a specific fMRI run. Thus, for each run PHONO or SEM, we included two regressors, PHONO (task) and Control-PHONO, and SEM (task) and Control-SEM, respectively. Each of them was convolved with a canonical hemodynamic response function (HRF). Movement parameters derived from the realignment corrections (three translations and three rotations) were included into the design matrix as additional factors of no interest. The GLM was then used to generate the parameter estimates of activity for each voxel, each condition and each participant. Statistical parametric maps were generated from the linear contrasts between the HRF parameter estimates for the four experimental conditions (i.e., task and control for each run). The spatial resolution of statistical parametric maps was the same as the spatial resolution of functional MR images (3 × 3 × 3.5 mm). The statistical analysis was performed at a first level (Individual level) by calculating the main contrasts that were PHONO (task) versus Control-PHONO and SEM (task) versus Control-SEM. These contrasts allowed us to identify language networks for phonology and semantic processes.

### ROI construction and extraction of the % MR signal (BOLD)

In accordance with our previously reported results from group analyses and based on previous literature on PHONO and SEM processing [2–4], we determined ten symmetrical frontal and temporal regions of interest (ROI), five in the left (LH) and five in the right hemisphere (RH). ROIs were defined based on the WFU PickAtlas toolbox (https://www.nitrc.org/projects/wfu_pickatlas/) from the Brodmann Area (BA) labeling. ROIs taken into account were, bilaterally, the inferior frontal gyrus *pars opercularis*, BA 44, *pars triangularis*, BA 45 and *pars orbitalis,* BA 47; middle temporal, BA 21 and superior temporal BA 22 gyri. For each ROI, each participant and each task (PHONO and SEM), the % of MR signal intensity variation (average of all voxels within a specific ROI) was measured. We defined 20 features for use in the ML classification approach (cf. 2.8)—the % of BOLD variation within the considered ROIs for PHONO (five ROI in the LH and five ROI in the RH) and for SEM (five ROI in the LH and five ROI in the RH).

### Machine learning

The ML approach aims to find a relationship between an input *X* = {*x*_1_, *x*_2_, …, *x*_*N*_} and an output $Y$. In our case, we inferred the relationship between the fMRI BOLD signal values and the participant condition (healthy; patient with epilepsy). In other words, we determined whether a participant is a patient with epilepsy or a healthy subject based on fMRI activation. More precisely, we aimed at determining the best combination(s) of features (according to region, hemisphere and task) showing the most predictive power in this binary classification. We used the XGBoost algorithm, an implementation of the gradient-boosted decision trees (GBDT) for this purpose. Assembly algorithms create and combine a high number of individually weak but complementary classifiers, to produce a robust estimator. This combination could be made in two ways: bagging (random forests) and boosting. The gradient boosting is built sequentially. Indeed, a new weak learner is constructed to be maximally correlated with the negative gradient of the loss function associated with the whole assembly for each iteration [36]. XGBoost belongs to the group of widely used tree learning algorithms [37]. A decision tree allows making prediction on an output variable based on a series of rules arranged in a tree-like structure. They consist of a series of split points, the nodes, in terms of the value of an input feature. The last node is a leaf and gives us the specific value of the output variable. Tree learning algorithms do not require linear features or linear interactions between features. They are significantly better classifiers than other algorithms (see [38]). Moreover, XGBoost, a type of gradient boosting, has two major improvements: (a) speeding up the tree construction and (b) proposing a new distributed algorithm for tree searching. All participants (samples) were described by the set of 20 features mentioned above. We combined these features into specific cognitively plausible subsets in order to reduce the number of combinations in the feature selection step (cf. 2.8.1). The entire procedure used in this study is presented in detail in *Supplementary Material.* We had eight missing among 1100 values (0.7%) from eight healthy participants for two features, BA 44 RH_SEM and the BA 44 RH_PHONO. We did not perform imputation or scaling on the data.

#### Feature selection method

The goal of feature selection was to choose a subset *X*_*S*_ of *X* that can predict *Y* with the best performance at minimal computational cost. Another objective was to gain insight into the underlying processes which generated the data.

There are three main categories of feature selection algorithms: filter, wrapper and embedded. In the present study, we have focused on the filter and wrapper methods. Filter methods are computed fast and provide a feature ranking in order to remove irrelevant features. Although some of them are multivariate, such as correlation-based feature selection (CFS), they do not involve a learning algorithm and can miss useful features. Wrapper approaches use a given classification algorithm for the evaluation of a specific subset of features by training and testing it with cross-validation. The space of all feature subsets is generated by the strategy defined above. An exhaustive search with these features involves around one million combinations $\left( { 2^{20} } \right)$. After evaluation, some filter methods (low variance, Fisher score, CFS, Laplacian score, spectral score) and the forward (SFS) and backward selection (SBS) wrapper methods were considered unsuccessful (i.e., with no feature subset stability and with no significance of the performance metrics). Filter methods were tested with scikit-feature Python libraries implementation [39]. SFS and SBS wrapper methods were performed with Mlxtend Python libraries [40].

Thus, we decided to perform another wrapper method, a reduced exhaustive search among a selection of 135 feature subsets. We did not perform an exhaustive search with the $2^{20}$ combinations of features, which would have been too long to compute and would have been prone to overfitting. These 135 different combinations of features were chosen and grouped in nine ‘thematic sets’ (see Table 2) based on ROI, hemisphere, and task. They are specified as follows: (a) three for SEM including a left hemisphere thematic set (with 15 subsets), a right hemisphere thematic set (including 15 subsets) and a bilateral thematic set (including 15 subsets); (b) three for PHONO including a left hemisphere thematic set (with 15 subsets), a right hemisphere thematic set (including 15 subsets) and a bilateral thematic set (including 15 subsets), and (c) three for SEM + PHONO including a left hemisphere thematic set (with 15 subsets), a right hemisphere thematic set (including 15 subsets) and a bilateral thematic set (including 15 subsets). These 135 feature subsets were combinations of the ROIs reflecting plausible patterns of language organization and reorganization based on literature results [3, 41, 42]. They varied according to ROIs (only frontal, only temporal or both), hemisphere (only left, only right or both hemispheres) and task (PHONO only, SEM only or PHONO + SEM).

**Table 2:** A total of 135 subsets were evaluated

Task	Subset	Fronto-temporal regions (FT)	Left hemisphere	Right hemisphere	Bilateral	
SEM only *or* PHONO only *or* SEM + PHONO	1	Partial F	BA 47LH	BA 47RH	BA 47LH	BA 47RH
	2	Partial F	BA 44LH, BA 45LH,	BA 44RH, BA 45RH,	BA 44LH, BA 45LH,	BA 44RH, BA 45RH,
	3	Total F	BA 44LH, BA 45LH, BA 47LH	BA 44RH, BA 45RH, BA 47RH	BA 44LH, BA 45LH, BA 47LH	BA 44RH, BA 45RH, BA 47RH
	4	Partial T	BA 21LH	BA 21RH	BA 21LH	BA 21RH
	5	Partial T	BA 22LH	BA 22RH	BA 22LH	BA 22RH
	6	Total T	BA 21LH, BA 22LH	BA 21RH, BA 22RH	BA 21LH, BA 22LH	BA 21RH, BA 22RH
	7	Partial FT	BA 21LH, BA 47LH	BA 21RH, BA 47RH	BA 21LH. BA 47LH	BA 21RH. BA 47RH
	8	Partial FT	BA 22LH, BA 47LH	BA 22RH, BA 47RH	BA 22LH, BA 47LH	BA 22RH, BA 47RH
	9	Partial FT	BA 21LH, BA 22LH, BA 47LH	BA 21RH, BA 22RH, BA 47RH	BA 21LH, BA 22LH, BA 47LH	BA 21RH, BA 22RH, BA 47RH
	10	Partial FT	BA 21LH, BA 44LH, BA 45LH	BA 21RH, BA 44RH, BA 45RH	BA 21LH, BA 44LH, BA 45LH	BA 21RH, BA 44RH, BA 45RH
	11	Partial FT	BA 22LH, BA 44LH, BA 45LH	BA 22RH, BA 44RH, BA 45RH	BA 22LH, BA 44LH, BA 45LH	BA 22RH, BA 44RH, BA 45RH
	12	Partial FT	BA 21LH, BA 22LH, BA 44LH, BA 45LH	BA 21RH, BA 22RH, BA 44RH, BA 45RH	BA 21LH, BA 22LH, BA 44LH, BA 45LH	BA 21RH, BA 22RH, BA 44RH, BA 45RH
	13	Partial FT	BA 21LH, BA 44LH, BA 45LH, BA 47LH	BA 21RH, BA 44RH, BA 45RH, BA 47RH	BA 21LH, BA 44LH, BA 45LH, BA 47LH	BA 21RH, BA 44RH, BA 45RH, BA 47RH
	14	Partial FT	BA 22LH, BA 44LH, BA 45LH, BA 47LH	BA 22RH, BA 44RH, BA 45RH, BA 47RH	BA 22LH, BA 44LH, BA 45LH, BA 47LH	BA 22RH, BA 44RH, BA 45RH, BA 47RH
	15	Total FT	BA 21LH, BA 22LH, BA 44LH, BA 45LH, BA 47LH	BA 21RH, BA 22RH, BA 44RH, BA 45RH, BA 47RH	BA 21LH, BA 22LH, BA 44LH, BA 45LH, BA 47LH	BA 21RH, BA 22RH, BA 44RH, BA 45RH, BA 47RH

Fifteen subsets were based on combinations of fronto-temporal (FT) regions according to hemisphere and task and defined as follows: (a) only frontal regions (partial subsets 1–2 and total subset 3); (b) only temporal regions (partial subsets 4–5 and total subset 6), and (c) combination of frontal and temporal regions (partial subsets 7–14 and total subset 15). These subsets were evaluated for three thematic sets according to task (semantic only, SEM only; phonological only, PHONO only; semantic and phonological combined, SEM + PHONO) and hemisphere (left hemisphere, right hemisphere and bilateral—both hemispheres)

#### Classification method

*The* Extreme Gradient Boosting (XGBoost) algorithm [43] was used for classification. It was implemented using the scikit-learn [40] Python libraries for all ML processes. Parameters for the algorithm were fixed (cf. Table 3) and not optimized by a grid search for the whole ML process (see [44]). Four parameters out of fifteen were set to particular values. The learning rate was typically set to 0.01 (default 0.3) as small values lead to much better generalization [45]. The number of boosted trees usually between some hundreds and thousands was set to 1200 (estimators). To prevent overfitting, the subsample was set to 0.7 (default 1). This added randomness and made the training robust to noise. The maximum depth of a tree was set to 3 (default 6) to reduce the model complexity.

**Table 3:** Results obtained for the selected subset SEM (semantic) LH (left hemisphere) BA 21 and BA47 in terms of AUC as the performance metric for each iteration of the outer MCCV, using the XGBoost algorithm (*n_estimators* = *1200, learning rate* = *0.01, subsample* = *0.7, max_depth* = *3*)

Iteration number	1	2	3	4	5	6	7	8	9	10	11	12
Subset selected	SEM L21 L47	SEM L21 L47	SEM L21 L47	SEM L21 L47	SEM L21 L47	SEM L21 L47	SEM L21 L47	SEM L21 L47	SEM L21 L47	SEM L21 L47	SEM L21 L47	SEM L21 L47
AUC (%)	93.75	87.50	87.50	93.75	93.75	93.75	100	83.33	83.33	93.75	87.50	100

#### Validation strategy

**Fig. 1:**
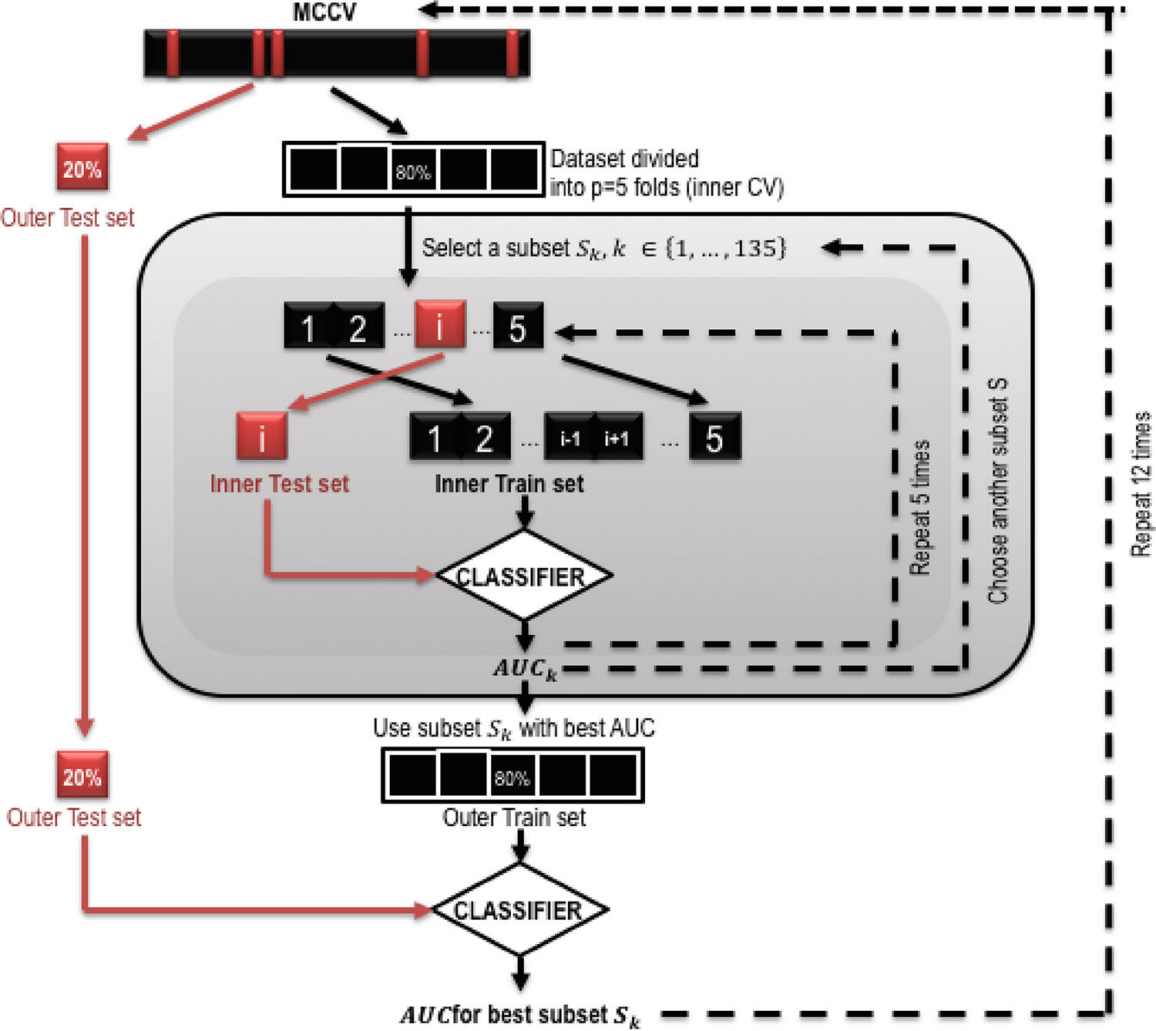
Illustration of the validation schema, using outer Monte Carlo cross-validation (MCCV)

The validation strategy is commonly used to prevent overfitting and to have a good assessment of model validity [44]. As illustrated in Fig. 1, we have used a nested cross-validation scheme with an outer Monte Carlo cross-validation (MCCV) (see [46, 47]), also called random subsampling, repeated twelve times in order to reduce variance and an inner *k*-fold cross-validation (*k*-fold CV) for feature selection with k = 5 (see [44, 45]). Feature selection must be done inside each training set of the outer cross-validation that estimates the performance of the model fitting approach [48]. First, we randomly select, without replacement, 80% of our data to form the training set. The remaining 20% was the validation set including a balanced number of epileptic and healthy subjects. Each participant appears in either the learning set or test set. Then fivefold cross-validation (CV) [49] was used only on the training set. It was split into five data blocks, four used for an inner training and the remaining one for the inner test. This was repeated five times by permuting the data blocks. The feature selection was done in this inner CV. Finally, the model was fitted on the training set with the feature subset that had the best performance and the prediction evaluated on the validation set that was held out from the feature selection step. All splits were performed in a stratified way to get the same ratio of patients with epilepsy. In order to assure stability of feature selection and get correct generalization of the classification performance, we repeated this process twelve times by randomly shuffling the dataset before splitting into training and validation sets. We finally obtained twelve performance metrics (cf. Table 3) for twelve feature subsets, based on a distribution of samples.

#### Metrics

The predictive power of a classifier was scored by the area under the receiver operating characteristic curve (AUC) as recommended by Provost [50–52]. The AUC can be interpreted as the probability that a classifier ranks a randomly chosen positive instance higher than a randomly chosen negative one (assuming ‘positive’ ranks higher than ‘negative’). The receiver operating curve (ROC) was the true positive rate plotted as a function of the false positive rate where the positive condition was to be a patient with epilepsy. It represents the performance of the model on a two-dimensional curve. The AUC value then reduces it to a number. A perfect model would score an AUC of 100% while a random classification would score 50%.

## Results

**Fig. 2:**
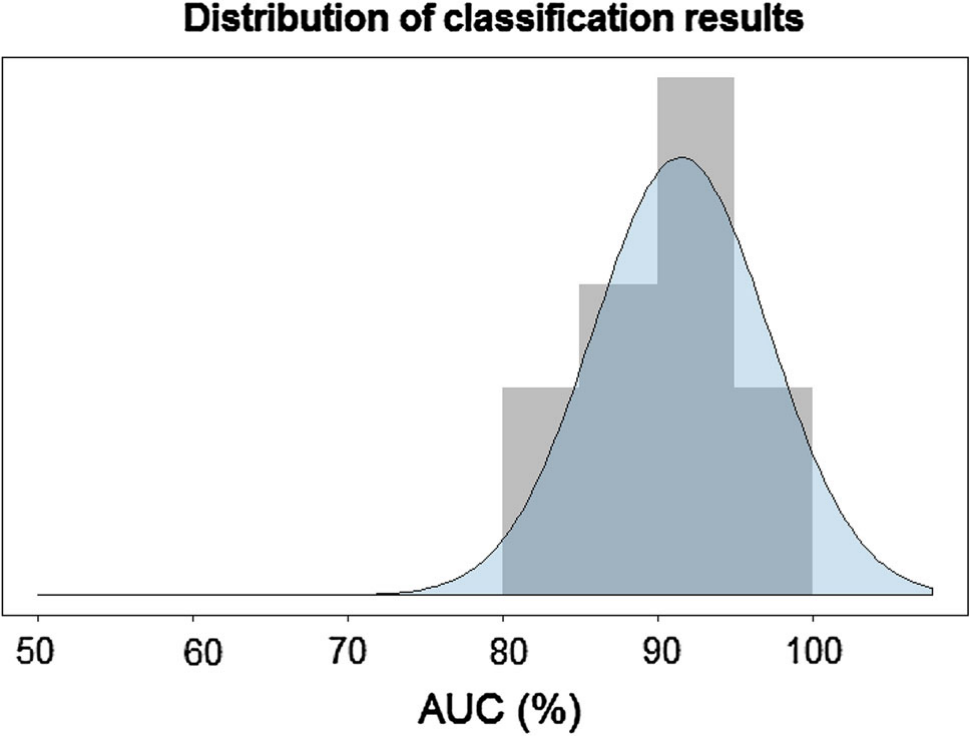
Distribution of the 12 AUC scores measured on the outer validation set of the Monte Carlo cross-validation (MCCV) around the mean score of 91%

As illustrated in Table 3 and Fig. 2, the feature subset *Semantic left hemisphere BA21_BA47* was selected each time among 135 candidates, with an AUC mean of 91 ± 5% on the validation set. The ML process showed a strong stability in feature selection and a very good level of classification performance.

## Discussion

This is a proof of concept study illustrating the ability of a specific ML approach, the XGBoost algorithm, to classify subjects in two distinct classes or categories, healthy/typical versus patients with epilepsy/atypical, according to their language representation, as determined with fMRI. This statistical method does not require manipulation of data and uses neurophysiological features reflecting the amount of activated language regions, for two main processes: semantic and phonological. Our results showed that a specific subset best distinguished the two categories of participants, namely the subset SEM_LH BA_47-21, showing that the left fronto-temporal activation induced by the SEM task was the most relevant to classify patients. This result can be discussed in the framework of current debates on language representation and reorganization in focal epilepsy [3]. Our result reflects reorganization of language networks in the predominant left hemisphere for language [4], and this can be considered as a specific ‘atypical’ profile of language representation. Indeed, the majority of individuals, mainly healthy, show ‘typical’ language representation with the left hemisphere predominant for language [4]. The majority of patients with focal epilepsy show higher variability of language representation within and between hemispheres, known as ‘atypical’ profiles, induced by the chronic development of the epileptic activity. Although many atypical profiles were described [42], three of them are more frequently observed (see [3]): (a) atypical inter-hemispheric representation with complete displacement of language areas from the left to the right regions [14, 53, 54]; (b) atypical inter-hemispheric representation with only partial displacement of language regions to the right hemisphere [17, 55]; and (c) atypical intra-hemispheric reorganization of language networks within the predominant, left hemisphere for language. This latter profile of reorganization fits well with our result, indicating that the best distinction between patients and healthy is based on changes occurring in the predominant left hemisphere for language. Indeed, an intra-hemispheric reorganization of language networks with supplementary or additional recruitment of fronto (BA47)—temporal (BA21) regions might occur in patients to maintain a correct level of language performance. The neurophysiological biomarker that seems to distinguish patients from healthy individuals at an intra-hemispheric level, is the activation of two crucial integrative regions—one frontal, the BA 47 and the other temporal, the BA 21—both responsible for semantic processing [56]. Their effect was located in the same left hemisphere, hence providing an intra-hemispheric biomarker of the distinction patients versus controls. Importantly, these regions should be considered in interaction rather than separately, given that they belong to neurocognitive models of language mainly for semantic processing, involved in retrieval, access, selection, online maintenance and activation of lexico-semantic representations [57]. Specifically, the BA 47 in the left inferior frontal gyrus is related to retrieval and selection of semantic features and supports controlled access to stored semantic representations [58]. The posterior middle temporal gyrus, BA 21, is generally implicated in the representation of verbal semantic information [59]. These two regions are anatomically and functionally connected. A reciprocal modulatory effect from the left inferior frontal gyrus (BA 47) to the left posterior middle temporal gyrus (BA 21) was shown by using dynamic causal modeling (DCM), suggesting top-down influences of the frontal cortex on the retrieval of semantic representations. In the opposite direction, the effective connectivity analyses also showed modulatory effects from the left BA 21 to the left BA 47, suggesting that posterior temporal regions provide relevant associations in verbal semantic memory to IFG for the purpose of retrieval [60]. In terms of anatomical connectivity, these regions are connected by white matter fibers such as the left inferior fronto-occipital fasciculus, left anterior thalamic radiation and left uncinate, and considered as the anatomical skeleton of the semantic network [61]. Overall, all these functional and anatomical data suggest that left fronto-temporal regions revealed by activation of BA 47 and BA 21, both part of the semantic network, are reorganized in patients with epilepsy compared to healthy subjects. Given that the majority of these patients show dysfunctions of temporal regions, this could explain why the semantic system is particularly sensitive, disrupted and reorganized in patients with epilepsy. An important contribution to this reorganization is added by the interaction between semantic language and memory processes, given that a part of the anatomical subjacent regions are common to both language and memory. This also explains why these two cognitive functions are increasingly examined together rather than separately [62]. These observations are reflected and confirmed by the neuropsychological testing, showing that these patients frequently have semantic (both language and memory) deficits. In conclusion, this biomarker of intra-hemispheric reorganization of fronto-temporal semantic networks revealed by the XGBoost algorithm for distinguishing patients from controls is in agreement with our knowledge on semantic processing. This result is in agreement with data from patients with epilepsy. It holds for the results obtained from invasive electrical stimulation [63] or noninvasive fMRI mapping [15, 64]. Specifically, patients with epilepsy show modification of language networks and they demonstrate a higher recruitment of the left hemispheric areas (inside and/or outside the ‘eloquent networks’) to ensure efficient language processing [64]. Nevertheless, compared to more visible inter-hemispheric profiles, the intra-hemispheric reorganization of language activity is more difficult to observe with classical fMRI statistical analyses. Mbwana et al. [64] suggested, for instance, that fMRI comparisons between patients and healthy subjects are constrained by a priori assumptions and reliance on preselection of cerebral regions and that the incidence of intra-hemisphere reorganization may be underestimated or masked. Based on these assumptions, we suggest that the ML–XGBoost algorithm could be a useful tool to detect the intra-hemispheric atypical reorganization patterns, more difficult to assess in patients, but having a major role in the neuroplasticity of language in patients with epilepsy. In terms of suitable tasks to map language networks, our results show that compared to phonological task, the semantic task is more reliable for classifying patients, even if both tasks activate fronto-temporal regions. The advantage of using a semantic task is that this task induces a more spread-out activation within frontal and temporal language networks. This assumption is in agreement with the findings by Billingsley et al. [9], showing that language reorganization in patients is mainly revealed by a semantic task, whereas a phonological task results in more specific prefrontal activation. Indeed the predictive capacity of postsurgical language outcome depends significantly on the regional location of brain activity. Moreover, a specific question raised by many investigations on language representation and lateralization is whether it is necessary to map the entire language system including frontal and temporal regions for this answer or whether only a partial mapping of frontal or temporal regions is sufficient to reveal the predominant hemisphere for language. Our winning subset SEM_LH BA_47-21 suggests that the robust classification of patients requires information on a larger fronto-temporal network which is efficiently revealed by a SEM task. In terms of clinical impact, we claim that the differential intra-hemispheric reorganization as reflected by SEM_LH BA_47-21 could suggest that left fronto-temporal regions are ‘sensitive-to-surgery’ and should be spared during surgery to avoid postsurgical language deficits. Methodologically, we claim that the XGBoost algorithm used in this study is able to compare cognitively plausible patterns (feature subsets) and highlight the best one, and able to separate categories of participants.

## Conclusions

The ML–XGBoost is a powerful statistical method of classification which detects nonlinear patterns in datasets with missing values. It shows significant potential for classifying patients with epilepsy based on the cerebral region, hemisphere and processing of their language representation. One subset, or a specific combination of features, the SEM_LH BA_47-21, was the most powerful, for identifying patients. The importance of this particular subset is plausible given the cognitive and clinical observations made with these patients.

## Electronic supplementary material

Below is the link to the electronic supplementary material.

Supplementary material 1 (DOCX 23 kb)
