# Supplementary material for: Machine learning–XGBoost analysis of language networks to classify patients with epilepsy
Source: Brain Inform. 2017 Apr 22;4(3):159–69. doi: 10.1007/s40708-017-0065-7 (PMC5563301; doi:10.1007/s40708-017-0065-7)
Supplement: Supplementary file 1 — Supplementary material 1 (DOCX 23 kb) [file 40708_2017_65_MOESM1_ESM.docx]

Supplementary Material for the manuscript: “Machine Learning-XGBoost Analysis of language networks to classify patients with epilepsy”

In order to achieve the Machine Learning analysis, we constructed this Python script code with the help of Scikit-Learn 0.18 python libraries (<http://scikit-learn.org>).

We wrote it with Pycharm 2016.2.3 (<https://www.jetbrains.com/pycharm/>) and we used Anaconda (<https://www.continuum.io/>) to get an update distribution of Python libraries.

*#-*- coding: utf-8 -*-
"""
=========================================
Nested cross-validation
=========================================
.. topic:: References:
 .. [1] `Cawley, G.C.; Talbot, N.L.C. On over-fitting in model selection and
 subsequent selection bias in performance evaluation.
 J. Mach. Learn. Res 2010,11, 2079-2107.
 <http://jmlr.csail.mit.edu/papers/volume11/cawley10a/cawley10a.pdf>`_
"""***import** timeit

**import** numpy **as** np
**import** pandas **as** pd
**import** xgboost **as** xgb

**from** sklearn.metrics **import** roc_auc_score
**from** sklearn.model_selection **import** StratifiedKFold, cross_val_score, train_test_split

**print**(__doc__)

*# Start time clock*start_time = timeit.default_timer()

*# Load data from CSV file*donnees = pd.read_csv**(./data.csv'**,sep=**';'**)

**print**(donnees.shape)

dataset = np.array(donnees)

*# Separate the data from the target attributes*X = dataset[:, 1:21]
y = dataset[:, 0]

*# Data balance***print**(np.count_nonzero(y == 1))
**print**(np.count_nonzero(y == 0))

*# Combinations of features to study

# Semantic Bilateral*S1 = [16, 18]
S2 = [8, 12, 10, 14]
S3 = [8, 12, 16, 10, 14, 18]
S4 = [0, 2]
S5 = [4, 6]
S6 = [0, 4, 2, 6]
S7 = [0, 16, 2, 18]
S8 = [4, 16, 6, 18]
S9 = [0, 4, 16, 2, 6, 18]
S10 = [0, 8, 12, 2, 10, 14]
S11 = [4, 8, 12, 6, 10, 14]
S12 = [0, 4, 8, 12, 2, 6, 10, 14]
S13 = [0, 8, 12, 16, 2, 10, 14, 18]
S14 = [4, 8, 12, 16, 6, 10, 14, 18]
S15 = [0, 4, 8, 12, 16, 2, 6, 10, 14, 18]

*# Phonologic Bilateral*P1 = [17, 19]
P2 = [9, 12, 11, 15]
P3 = [9, 12, 17, 11, 15, 19]
P4 = [1, 3]
P5 = [5, 7]
P6 = [1, 5, 3, 7]
P7 = [1, 17, 3, 19]
P8 = [5, 17, 7, 19]
P9 = [1, 5, 17, 3, 7, 19]
P10 = [1, 9, 12, 3, 11, 15]
P11 = [5, 9, 12, 7, 11, 15]
P12 = [1, 5, 9, 12, 3, 7, 11, 15]
P13 = [1, 9, 12, 17, 3, 11, 15, 19]
P14 = [5, 9, 12, 17, 7, 11, 15, 19]
P15 = [1, 5, 9, 12, 17, 3, 7, 11, 15, 19]

*# SEM+PHONO Bilateral*M1 = [16, 18, 17, 19]
M2 = [8, 12, 10, 14, 9, 13, 11, 15]
M3 = [8, 12, 16, 10, 14, 18, 9, 13, 17, 11, 15, 19]
M4 = [0, 2, 1, 3]
M5 = [4, 6, 5, 7]
M6 = [0, 4, 2, 6, 1, 5, 3, 7]
M7 = [0, 16, 2, 18, 1, 17, 3, 19]
M8 = [4, 16, 6, 18, 5, 17, 7, 19]
M9 = [0, 4, 16, 2, 6, 18, 1, 5, 17, 3, 7, 19]
M10 = [0, 8, 12, 2, 10, 14, 1, 9, 13, 3, 11, 15]
M11 = [4, 8, 12, 6, 10, 14, 5, 9, 13, 7, 11, 15]
M12 = [0, 4, 8, 12, 2, 6, 10, 14, 1, 5, 9, 13, 3, 7, 11, 15]
M13 = [0, 8, 12, 16, 2, 10, 14, 18, 1, 9, 13, 17, 3, 11, 15, 19]
M14 = [4, 8, 12, 16, 6, 10, 14, 18, 5, 9, 13, 17, 7, 11, 15, 19]
M15 = [0, 4, 8, 12, 16, 2, 6, 10, 14, 18, 1, 5, 9, 13, 17, 3, 7, 11, 15, 19]

*# Semantic Left Hemisphere*I1 = [16]
I2 = [8, 12]
I3 = [8, 12, 16]
I4 = [0]
I5 = [4]
I6 = [0, 4]
I7 = [0, 16]
I8 = [4, 16]
I9 = [0, 4, 16]
I10 = [0, 8, 12]
I11 = [4, 8, 12]
I12 = [0, 4, 8, 12]
I13 = [0, 8, 12, 16]
I14 = [4, 8, 12, 16]
I15 = [0, 4, 8, 12, 16]

*# Phonologic Left Hemisphere*J1 = [17]
J2 = [9, 13]
J3 = [9, 13, 17]
J4 = [1]
J5 = [5]
J6 = [1, 5]
J7 = [1, 17]
J8 = [5, 17]
J9 = [1, 5, 17]
J10 = [1, 9, 13]
J11 = [5, 9, 13]
J12 = [1, 5, 9, 13]
J13 = [1, 9, 13, 17]
J14 = [5, 9, 13, 17]
J15 = [1, 5, 9, 13, 17]

*# Semantic Right Hemisphere*K1 = [18]
K2 = [10, 14]
K3 = [10, 14, 18]
K4 = [2]
K5 = [6]
K6 = [2, 6]
K7 = [2, 18]
K8 = [6, 18]
K9 = [2, 6, 18]
K10 = [2, 10, 14]
K11 = [6, 10, 14]
K12 = [2, 6, 10, 14]
K13 = [2, 10, 14, 18]
K14 = [6, 10, 14, 18]
K15 = [2, 6, 10, 14, 18]

*# Phonologic Right Hemisphere*L1 = [19]
L2 = [11, 15]
L3 = [11, 15, 19]
L4 = [3]
L5 = [7]
L6 = [3, 7]
L7 = [3, 19]
L8 = [7, 19]
L9 = [3, 7, 19]
L10 = [3, 11, 15]
L11 = [7, 11, 15]
L12 = [3, 7, 11, 15]
L13 = [3, 11, 15, 19]
L14 = [7, 11, 15, 19]
L15 = [3, 7, 11, 15, 19]

*# SEM+PHONO Left Hemisphere*N1 = [16, 17]
N2 = [8, 12, 9, 13]
N3 = [8, 12, 16, 9, 13, 17]
N4 = [0, 1]
N5 = [4, 5]
N6 = [0, 4, 1, 5]
N7 = [0, 16, 1, 17]
N8 = [4, 16, 5, 17]
N9 = [0, 4, 16, 1, 5, 17]
N10 = [0, 8, 12, 5, 9, 13]
N11 = [4, 8, 12, 5, 9, 13]
N12 = [0, 4, 8, 12, 1, 5, 9, 13]
N13 = [0, 8, 12, 16, 1, 9, 13, 17]
N14 = [4, 8, 12, 16, 5, 9, 13, 17]
N15 = [0, 4, 8, 12, 16, 1, 5, 9, 13, 17]

*# SEM+PHONO Right Hemisphere*R1 = [18, 19]
R2 = [10, 14, 11, 15]
R3 = [10, 14, 18, 11, 15, 19]
R4 = [2, 3]
R5 = [6, 7]
R6 = [2, 6, 3, 7]
R7 = [2, 18, 3, 19]
R8 = [6, 18, 7, 19]
R9 = [2, 6, 18, 3, 7, 19]
R10 = [2, 10, 14, 7, 11, 15]
R11 = [6, 10, 14, 7, 11, 15]
R12 = [2, 6, 10, 14, 3, 7, 11, 15]
R13 = [2, 10, 14, 18, 3, 11, 15, 19]
R14 = [6, 10, 14, 18, 7, 11, 15, 19]
R15 = [2, 6, 10, 14, 18, 3, 7, 11, 15, 19]

*# Subsets*subsets_S = [S1, S2, S3, S4, S5, S6, S7, S8, S9, S10, S11, S12, S13, S14, S15]
subsets_P = [P1, P2, P3, P4, P5, P6, P7, P8, P9, P10, P11, P12, P13, P14, P15]
subsets_M = [M1, M2, M3, M4, M5, M6, M7, M8, M9, M10, M11, M12, M13, M14, M15]
subsets_I = [I1, I2, I3, I4, I5, I6, I7, I8, I9, I10, I11, I12, I13, I14, I15]
subsets_J = [J1, J2, J3, J4, J5, J6, J7, J8, J9, J10, J11, J12, J13, J14, J15]
subsets_K = [K1, K2, K3, K4, K5, K6, K7, K8, K9, K10, K11, K12, K13, K14, K15]
subsets_L = [L1, L2, L3, L4, L5, L6, L7, L8, L9, L10, L11, L12, L13, L14, L15]
subsets_L = [L1, L2, L3, L4, L5, L6, L7, L8, L9, L10, L11, L12, L13, L14, L15]
subsets_N = [N1, N2, N3, N4, N5, N6, N7, N8, N9, N10, N11, N12, N13, N14, N15]
subsets_R = [R1, R2, R3, R4, R5, R6, R7, R8, R9, R10, R11, R12, R13, R14, R15]


subsets_sum = subsets_S + subsets_P + subsets_M + subsets_I + subsets_J + subsets_K + subsets_L + subsets_N + subsets_R

*##############################################
# Statistical Learning
##############################################

# XGBoost hyperparameters*model_base = xgb.XGBClassifier(max_depth=3, subsample=0.7, learning_rate=0.01, n_estimators=1200)

value=[]

*# Twelve random sates randomly choosen for the outer-MCCV***for** i **in** [32,41,45,52,65,72,96,97,112,114,128,142]:

 **print** (**'Random state : '**, i)

 *# Split the dataset into two stratified parts, 80% for Outer training set* X_train, X_test, y_train, y_test = train_test_split(X, y, train_size=0.8, random_state=i, stratify=y)

 *# Choose k-fold cross-validation technique for the inner loop* inner_cv = StratifiedKFold(n_splits=5, shuffle=True, random_state=i)

 *# Set temporary variables* best_subset = []
 best_auc = -np.inf

 *# Loop over the 135 features combinations* **for** subset **in** subsets_sum:

score = cross_val_score(model_base, X=X_train[:,subset], y=y_train, cv=inner_cv, scoring=**'roc_auc'**)
 **if** score.mean() > best_auc:
 best_auc = score.mean()
 best_subset = subset

*# Train the model on the Outer training set with the selected feature combination*
 model_base.fit(X_train[:,best_subset], y_train)

*# Calculate the predicted labels with the model on the Outer test set with the selected feature combination*
 y_pred = model_base.predict(X_test[:,best_subset])

*# Calculate the AUC of the ROC curve between predicted and true labels*
 auc = roc_auc_score(y_test, y_pred)

 **print**(**'Selected features:'**, best_subset,**'; Outer Test AUC: '**,auc)

 value.append(auc)

*# Evaluation of the mean and standard deviation of the twelve AUC*
score = np.asarray(value)
**print "Twelve times repeated Outer Test AUC : %1.2f"** % score.mean(), **"; %1.2f"** % score.std()

**print 'Time execution : '**, timeit.default_timer() - start_time
